# *SYNCmoss* software package for fitting Mössbauer spectra measured with a synchrotron Mössbauer source

**DOI:** 10.1107/S1600577523001686

**Published:** 2023-03-31

**Authors:** Sergey Yaroslavtsev

**Affiliations:** a ESRF– The European Synchrotron, CS40220, 38043 Grenoble Cedex 9, France; Paul Scherrer Institut, Switzerland

**Keywords:** synchrotron Mossbauer source, software, fitting

## Abstract

*SYNCmoss* is a software package dedicated to fitting Mössbauer spectra measured with a synchrotron Mössbauer source (SMS). Operation conditions of an SMS can be continuously varied from high resolution and low intensity to the opposite extreme. These variations influence the width and shape of the instrumental function. The main purpose of *SYNCmoss* is to fit data obtained with instrumental functions of various shapes. *SYNCmoss* allows the instrumental function to be extracted from the spectrum of a standard absorber, and then to utilize it in the fitting procedures for the samples studied. *SYNCmoss* is standalone software – it does not require installations of any software environment. Setting input parameters and the fitting procedure are controlled within a graphical user interface. High-intensity mode of the SMS leads to a short acquisition time, allowing for fast (for example, *operando*) measurements resulting in a long sequence of spectra with close or smoothly varying parameters. For such cases, *SYNCmoss* provides an option to fit a series of spectra in automatic mode. *SYNCmoss* provides fitting models for various cases of hyperfine interaction including the full Hamiltonian for the combined interaction. Finally, for generality, the software has the ability to also fit conventional Mössbauer spectra and simple cases of nuclear forward scattering spectra.

## Abstract 2

*SYNCmoss* is software which allows to fit Mössbauer spectra measured with a synchrotron Mössbauer source taking into account the shape of instrumental function. The software provides a comprehensive functionality to fit data within various static and relaxation models along with parameter distributions and correlations.

## 1. Introduction

A synchrotron Mössbauer source (SMS) enables conventional (energy-domain) Mössbauer spectroscopy for the ^57^Fe isotope at synchrotron radiation facilities (Smirnov *et al.*, 1997 ▸; Mitsui *et al.*, 2007 ▸; Potapkin *et al.*, 2012 ▸). An SMS provides several significant advantages: (1) small beam size, (2) almost 100% recoilless radiation, and (3) polarized radiation, making it extremely useful in different scenarios where conventional Mössbauer spectroscopy cannot be used or its usability is noticeably limited, like at high pressures (Potapkin *et al.*, 2013 ▸; Kupenko *et al.*, 2019 ▸; Hamada *et al.*, 2021 ▸), with small inclusions (Andrault *et al.*, 2018 ▸; Nestola *et al.*, 2016 ▸; Blukis *et al.*, 2017 ▸), for surface studies (Cini *et al.*, 2018 ▸; Fujiwara *et al.*, 2021 ▸; Mitsui *et al.*, 2020 ▸), and others.

In comparison with radioactive sources, the SMS can be adjusted for specific experiments. This adjustment includes changes in the intensity and in the shape of the instrumental function to achieve maximum signal-to-noise ratio (Yaroslavtsev & Chumakov, 2022 ▸). In order to fit SMS spectra, one should take into consideration the exact shape of the instrumental function in each individual case. The influence of the instrumental function becomes more pronounced at higher statistics, where features of its shape could not be attributed to the noise and, thus, will lead to uncertainties in the interpretation of the results. Therefore, the instrumental function should be determined prior to any fitting. Most of the software are aimed to fit spectra measured by conventional Mössbauer spectroscopy [see, for example, Rusakov & Matsnev (2012 ▸)] and therefore there is no need to take into account the shape of the instrumental function. Only few software solutions provide the ability to somehow approximate the instrumental function [see, for example, Prescher *et al.* (2012 ▸) and Žák & Jirásková (2006 ▸)]. In one of the most popular pieces of software, *MossA* (Prescher *et al.*, 2012 ▸), it can be approximated by a Lorentzian, Gaussian or Lorentzian-squared shape, but actually the instrumental function of SMS is an asymmetric function, the shape of which cannot be described analytically (Smirnov *et al.*, 2011 ▸; Yaroslavtsev & Chumakov, 2022 ▸). The main aim of *SYNCmoss* is to provide the ability to determine the instrumental function and to use it for fitting of experimental spectra. This feature could be crucial in cases when spectra contain both high- and low-intensity subspectra, or when the determination of hyperfine parameters with a high accuracy is required (corresponding examples are discussed below).

## 2. Instrumental function

The instrumental function should be found prior to the fitting session, as it is used in all fitting procedures. It can be extracted from the spectrum of a standard absorber, the intrinsic spectrum of which is known^1^ [see Yaroslavtsev & Chumakov (2022 ▸) for how to extract an intrinsic spectrum]. Unfortunately, the instrumental function shape cannot be described by an analytical equation, which is why it is fitted with a number of Gaussian lines (the exact number can be changed by the user). The Gaussian form was chosen due to its steeper profile compared with a Lorentzian or Lorentzian-squared – therefore fitting the latter two with a set of Gaussians is easier than vice versa. After the extracting procedure is completed, the instrumental function found will be used in all further fittings.

**Figure 1:**
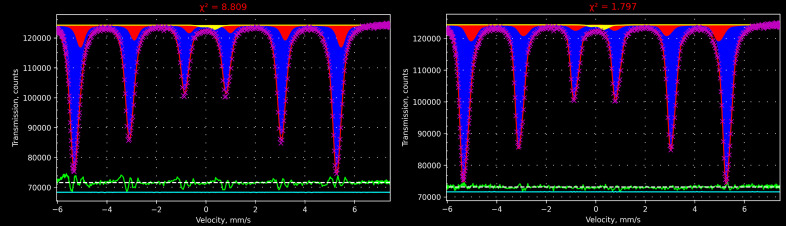
Spectrum of an α-iron foil measured with SMS and the result of fitting with an instrumental function of Lorentzian-squared shape (left) and with an instrumental function extracted from a spectrum of the standard sample (right). The model consists of a sextet related to α-Fe (blue subspectrum), an additional sextet with a smaller magnetic field typical of the presence of residual traces of other metals (red subspectrum), and an asymmetric doublet for the Fe in Be lenses (yellow subspectrum). The green line under the spectra is the residual line. The cyan line shows the quality of the numeric integration (see Section 5 below for more details). The result of fitting is presented in the same way as in the GUI of *SYNCmoss*.

Fig. 1 ▸ shows the SMS spectrum of an α-iron foil fitted with the instrumental function extracted from the standard absorber, and also with the Lorentzian-squared shape instrumental function, the width of which was also determined from the spectrum of the standard absorber. The difference between approaches is clearly seen in the resulting χ^2^ (Pearson’s criteria) and in the residual lines. Fitting with the incorrect instrumental function provides different hyperfine parameters. To reach an acceptable χ^2^ it will require more subspectra which are not physically justified, thus it could create uncertainties in the interpretation of the results. Utilizing the instrumental function extracted from the standard absorber, the hyperfine parameters of the second component are the following: central shift δ = −0.01 (1) mm s^−1^, quadrupole shift ε = 0.00 (1) mm s^−1^, and hyperfine magnetic field *H* = 31.0 (1) T, and the effective thicknesses of the two components are 7.95 (5) and 0.59 (4). These parameters are reasonable and show that the second component corresponds to Fe atoms that have some other metal atom in the nearest environment (a small impurity of other transition metals is typical for α-iron foils). At the same time, the Lorentzian-squared shape for the instrumental function results in the second component having δ = 0.19 (3) mm s^−1^, ε = 0.02 (2) mm s^−1^ and *H* = 32.6 (1) T, and the effective thicknesses for the two components are 7.8 (1) and 0.81 (7). Most importantly, the fitting quality drops down significantly – χ^2^ is equal to 8.8 or worse^2^ compared with 1.8. Thereby, the second component could be incorrectly interpreted as a presence of Fe oxides on the surface because the central shift is close to typical values for Fe^3+^. Such a mistake occurs because the second subspectrum matches features of the main component caused by an asymmetric instrumental function. Indeed, in this case (Lorentzian-squared shape of the instrumental function) the position of the resonance lines of the second subspectrum are shifted to the right of the main subspectrum (see Fig. 1 ▸, left), describing actually the features of the instrumental function. On the contrary, with a correct instrumental function the resonance lines of the second subspectrum are closer to the center indicating a smaller value of the hyperfine magnetic field. Of course, hyperfine parameters of the dominant component coincide with theory (δ = 0.00 mm s^−1^, ε = 0.00 mm s^−1^, *H* = 33.04 T) within error in both cases. In practice, spectra of higher complexity will create more uncertainties and imperfections due to incorrect instrumental functions.

The standard absorber was measured several times under SMS conditions close to standard [temperature of iron borate around 75.825°C and incidence angle of 0.004°; see Yaroslavtsev & Chumakov (2022 ▸) for more details]. This is the most typical mode of SMS operation. Under these conditions the instrumental function looks like a single line but has some asymmetric features. Extraction of the instrumental function from these spectra shows that three Gaussian lines are already sufficient to approximate the shape of the instrumental function. Of course, this depends on the quality (*i.e.* the signal-to-noise ratio) of the standard absorber spectrum: the higher the quality, the larger the number of Gaussians required. The quality of the tested spectra was up to 180. The quality of the standard absorber spectrum should not be less than the quality of the spectra of the studied samples, otherwise an error induced in the process of determining the instrumental function could prevail over a standard deviation of the found optimal parameters for the experimental spectrum. A change of the SMS conditions also could result in a more complex instrumental function and the necessity for more Gaussian lines to approximate the instrumental function.

In order to make this software more generalized there is also an option to fit conventional Mössbauer spectra (CMS). When fitting spectra measured with a radioactive source, the Voigt shape is a reasonable approximation of the instrumental function. However, its width could vary from source to source. The Lorenzian width is close to the natural width and the Gaussian width is usually about 0.06 mm s^−1^ which is set as a default value, but if it is known specifically it can be set manually in the appropriate text box in the GUI. After setting the instrumental function, the fitting procedure is the same for the CMS and the SMS cases.

## 3. Models

Fitting of Mössbauer spectra starts with choosing the model and then finding its optimal parameters to match the experimental spectrum. A model can consist of submodels which describe specific cases of hyperfine interaction. The more models of different hyperfine interactions that are in the software, the more flexible tool it becomes. *SYNCmoss* provides to the user a decent set of models which will be described in this section (and which are implemented in the software at the time of publishing this work). In particular, the software contains one of the most general cases of the full Hamiltonian describing the combined interaction in a most general case where the asymmetry parameter is not necessary zero. Since implementation of the model for the SMS and CMS is almost the same, all models are present for both of them, but not for the case of nuclear forward scattering (NFS) which is standalone.

Each model includes the unitless absorber effective thickness as a parameter. Each submodel corresponds to Fe atoms in a specific local environment, and an effective thickness represents its fraction more accurately than a subspectrum area. This could be crucial in cases when saturation effects are significant (usually this takes place with an effective thickness of ≳2). The effective thickness, *T*, is

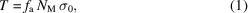

where *f*_a_ is the probability of recoilless absorption, *N*_M_ is the number of Mössbauer nuclei per unit area of the absorber, and σ_0_ is the resonant cross section. The effective thickness can be estimated before fitting by simple calculations if sample information is known.

### 3.1. Basic models

Most Mössbauer experimental spectra can be fitted using only simple models which approximate a solution to the hyperfine interactions static Mössbauer problem. Such models work only under specific conditions, which, however, are rather common.

Even though simple cases can be described by solving the full Hamiltonian in general terms, it may be overcomplicated and inconvenient to use such a representation. In this regard, *SYNCmoss* contains classical approximations which describe the hyperfine interaction in the presence of only an electric monopole interaction (singlet), when there is an additional electric quadrupole interaction (doublet), and the case when there is also a magnetic dipole interaction which is significantly larger than the electrical quadrupole interaction (sextet).

The singlet, doublet and sextet models contain parameters defining the isomer shift, and the widths of the deconvoluted Lorentzian and Gaussian functions which define the Voigt shape of a resonance line. The doublet also includes a quadrupole shift (half of a quadrupole splitting), the ratio between line intensities and the ratio between line widths. The first ratio is needed to describe polarization effects and texture, and the second is helpful when there is a linear correlation between an isomer shift and a quadrupole shift, as often observed in glasses (Dunlap *et al.*, 1998 ▸); the sextet, in addition, includes a hyperfine magnetic field, width of hyperfine magnetic field normal distribution, ratios between line intensities (*I*_1_:*I*_2_ and *I*_1_:*I*_3_, where *I*_*n*_ is the intensity of the *n*th line) and two parameters which provide additional shifts of the resonance line positions for a more accurate approximation of the combined interaction [for more details see Onodera *et al.* (1987 ▸)].

### 3.2. Combined interaction/full Hamiltonian

In cases where approximate models do not fit the experimental spectra, one can use the exact full solution to the general combined interactions static Mössbauer problem. This is also called the full Hamiltonian model because it requires the full Hamiltonian of the combined interaction to be diagonalized and its eigenvectors and eigenvalues to be found. This model is indispensable if the energies of the electric quadrupole and magnetic dipole interactions are comparable. In such cases line positions and intensities do not match a simple sextet, and sometimes even two additional lines become visible.

The full Hamiltonian model in *SYNCmoss* is based on the approach presented by Voyer & Ryan (2006 ▸) where this model is described in detail for the CMS case. The coordinate system is chosen to match eigenvectors of the electric field gradient tensor, and the orientation of the main component *V*_*zz*_ is chosen as the quantization axis. In *SYNCmoss* the multipolarity of transitions is assumed to be pure M1, because the E2 contribution is negligible in the case of ^57^Fe [less than 0.1%; see Bhat (1998 ▸) and references therein]. There are two Hamiltonian models in this software, one for a powder sample and another for a single crystal. It is obvious that powder sample solutions for SMS and CMS are completely the same due to an all-around averaging. In the case of a single crystal, the switch from CMS to SMS is achieved by changing the amplitude angular distributions^3^ [equations (20), (21) and (22) of Voyer & Ryan (2006 ▸)],

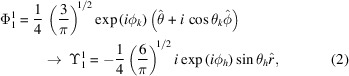




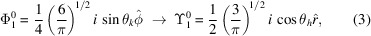




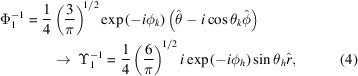

where θ_*k*_ and ϕ_*k*_ are used for the CMS describing the direction of a γ-quantum propagation, and θ_*h*_ and ϕ_*h*_ are for the SMS defining a direction of the magnetic vector of the beam.

### 3.3. Relaxations

In addition to static models, the *SYNCmoss* package includes two common relaxation models. They represent dynamic cases when changes of a local state happen on the time scale of the Mössbauer window (the nucleus excited state lifetime).

The two-state relaxation model is based on the Blume approach (Blume & Tjon, 1968 ▸), but describes the more general case when an isomer shift and a quadrupole shift can be different for two states. This generalization is straightforward because a relaxation appears between each pair of lines corresponding to the transition between two specific levels (for example, 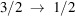 ), thus it only requires implementation of a different isomer shift, quadrupole shift and hyperfine field for each state. This model can be useful in cases of two-paramagnetic-state relaxation which can be observed in materials with a superstructure or with movable ions [see, for example, Ellis *et al.* (2006 ▸)] and as an approximation of a fast fluctuating hyperfine magnetic field as in the initial model (Blume & Tjon, 1968 ▸). This model can be written in the analytic way only for the case of zero Gaussian broadening (an ideal homogeneous sample). Thus, Gaussian broadening in this model is added afterwards in a numeric way by point-by-point convolution^4^.

The many-state magnetic relaxation model is described by Jones & Srivastava (1986 ▸). This model is implemented without changes. It is dedicated to fitting of the superparamagnetic relaxation. However, it also could be useful in cases when some small areas containing magnetic ions are spread within one bigger particle (Yaroslavtsev *et al.*, 2020 ▸).

### 3.4. Distribution

There are many cases of the hyperfine interaction that can be described (or approximated) in terms of a distribution and correlations of some parameters within a simpler model. Examples are various relaxations, spin density waves, cases of correlated parameters distributions induced by inhomogeneity, and others.

To deal with such cases, *SYNCmoss* provides an ability to distribute one of the parameters within any of the above-described models. The distribution is set by left and right boundaries for a distributed parameter and an equation for the density probability function which could be written in a simple mathematical language and could contain parameters from other models or independent variables. The number of points for this distribution can also be set by the user. A low number of points does not represent a real distribution, but increasing the number of points, of course, increases the calculation time. Therefore, a compromise should be found for each specific case. Other parameters can correlate with the distributed one; in this case the user is able to set functions which describe the dependencies of these parameters on the distributed one.

#### 3.4.1. Distribution example

Here we consider one example. In order to resolve the magnetic properties of a sample it could be useful to perform measurements under an external magnetic field. If the studied sample is a powder, then there is a distribution of particle orientations and thus anisotropy axis orientations, while orientations of the external magnetic field and radiation are fixed. Therefore, this will lead to a two-dimensional distribution of the hyperfine magnetic field and its orientation relative to the magnetic vector of the beam (Long *et al.*, 2011 ▸). Here we will consider one of the most common cases where the absolute values of the anisotropy energy and exchange integral are significantly larger than the interaction of magnetic moments with an external field (hard magnetic material) and easy axis anisotropy.

**Figure 2:**
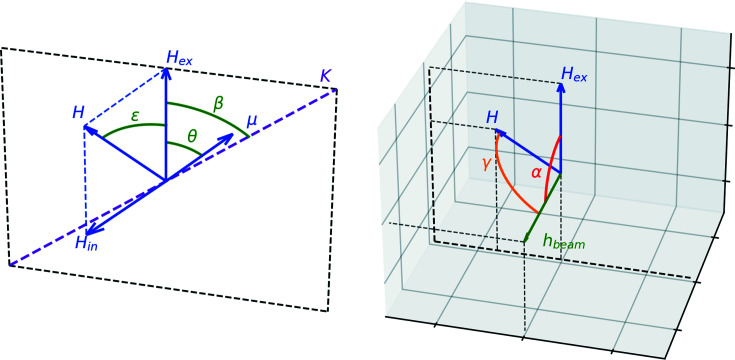
Directions of the magnetic moment (μ), anisotropy axis (*K*), external magnetic field (*H*_ex_), internal hyperfine field (*H*_in_), full hyperfine field (*H*), and magnetic vector of the beam (*h*_beam_) for a certain orientation of the magnetic moment. All vectors except *h*_beam_ are coplanar (left panel). The directions of *h*_beam_ and *H*_ex_ depend on the experimental setup and are predefined, while *H* could be different for each orientation of the magnetic moment (right panel).

First we need to determine the direction of the magnetic moments. For a specifically oriented particle, this can be found by minimization of the magnetic energy,

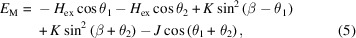

where *H*_ex_ is the external magnetic field, *K* is the anisotropy constant multiplied by a particle volume and divided by the magnetic moment, *J* is the exchange integral divided by the magnetic moment and by the number of neighbors, β is the angle between the anisotropy axis and the external magnetic field, and θ_1_ and θ_2_ determine the angles between magnetic moments and the external magnetic field. Two different angles (θ_1_ and θ_2_) are needed for the antiferromagnetic case. After finding sets of values for θ_1_ and θ_2_ they could be united into one set which we will call θ. Fig. 2 ▸ shows all angles and vectors for one of the magnetic moments in a particle with a certain orientation.

The internal hyperfine magnetic field in most cases is collinear (and usually also opposite) to the magnetic moment of the Fe atom. Only two impacts due to the full hyperfine field are non-parallel to the magnetic moment – an external field and dipole–dipole interaction. A dipole–dipole interaction is usually two orders of magnitude less than a full hyperfine field, thus for most cases we can assume its perpendicular component to be negligible. Thus, knowing the orientation of the magnetic moment one could find the full hyperfine magnetic field,

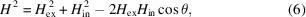

where *H*_in_ is an intrinsic hyperfine magnetic field and θ is some specific value from sets of θ_1_ or θ_2_.

The intensities of the lines in a sextet depend on the angle γ between the magnetic vector of the beam (*h*_beam_) and the full hyperfine magnetic field (*H*)^5^,

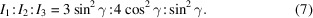

Hereby, we have sextets with a distribution of the hyperfine field and a distribution of γ which defines the intensities of the resonance lines. To build a model we need to find the distribution of the effective thickness over the hyperfine field, *T*(*H*) (which is a probability density function of *H* multiplied by the full effective thickness), and the distribution of γ as a function of *H*.

Let us start from the γ problem. We need to introduce the angle α between an external magnetic field and magnetic vector of the beam (see Fig. 2 ▸). This angle should be known from the experimental setup, so we consider it a constant. To find γ we need also the angle ε between an external magnetic field and the full magnetic field (see Fig. 2 ▸), which can be found from the equation

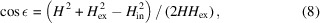

where ε corresponds to a set of particles with the same absolute value of *H* but differently oriented in space (rotation around *H*_ex_). If *H* = 0 then ε does not matter because there will be no magnetic splitting; if *H*_ex_ = 0, then ε, θ and α should be redefined as angles between some chosen direction (instead of external field) and corresponding vectors.

Because all sextets which correspond to specific ε have the same splitting but different ratio of line intensities, we can find the average for 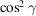 for specific ε,

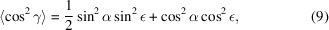

and finally the intensities of the lines in our model are 

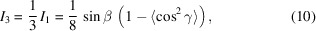




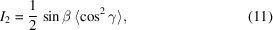

where 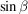 appears as a probability density function of β. The use of the 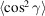 value is not an approximation but provides an exact solution because the averaging is performed for a specific value of a full hyperfine field (and so for a specific β). As seen from the equations, the ratio of the line intensities does not depend on β. Thus, the problem can be reduced to a much simpler one, namely to a correlated distribution of *H* and 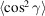 [in other words *I*_1_:*I*_2_:*I*_3_(*H*)].

In the general case, *T*(*H*) could be found from the following idea. Due to the particle orientation along with the direction of anisotropy being randomly distributed, β has a probability density function equal to 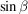 . θ is a function of β, while *H* is a function of θ. Thus, knowing *H*(β) and the distribution of β and the full effective thickness, we are able to find *T*(*H*). *T*(*H*) along with *I*_1_:*I*_2_:*I*_3_(*H*) define the position and intensities of the lines, together with the central shift and quadrupole splitting which are not distributed.^6^

In the case of hard magnetic material it is obvious that θ_1_ and θ_2_ will approach β and π−β if *J* < 0 (antiferromagnetic) or β and −β if *J* > 0 (ferromagnetic). It is easy to show that the probability density function of the full hyperfine field will be linear with a zero intercept in the range from 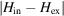 to *H*_in_ + *H*_ex_ if *J* < 0 or to 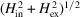 if *J* > 0 and equal to zero out of this range.

**Figure 3:**
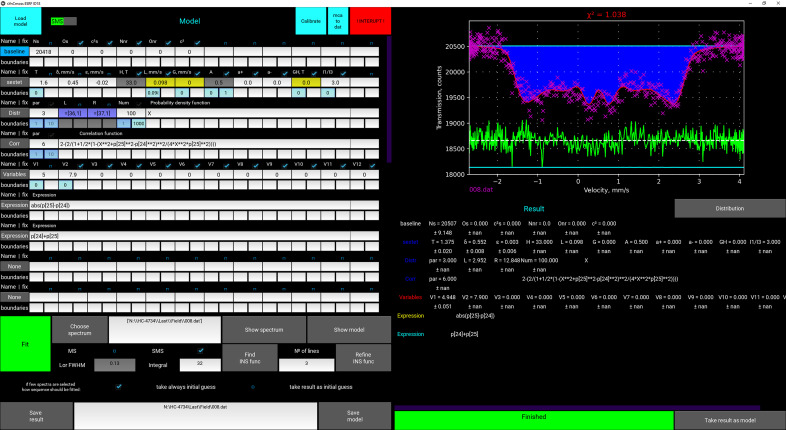
Example of data fitting for a powder antiferromagnetic sample (SmFeAsO) at 5 K under an external magnetic field of 7.9 T oriented perpendicular to the magnetic vector of the beam. Fitting was performed utilizing the model described in Section 3.4.1. The green line is a residual between the experimental spectrum and the model. The cyan line is the difference between a model calculated with a selected number of points per integration and that with four times more points.

Fig. 3 ▸ shows an example of an experimental spectrum of hard magnetic material. Fitting was performed within the approach described above using a linear distribution of the hyperfine field and correlated ratio of line intensities. This is an example where such distributions with correlations allow users to create new models without changes in code.

### 3.5. Nuclear forward scattering

In addition, *SYNCmoss* also provides models for simple cases of hyperfine interaction (singlet, doublet, sextet) to fit ^57^Fe time-domain Mössbauer spectra which can be measured by the nuclear forward scattering (NFS) technique. This makes *SYNCmoss* more comprehensive software for synchrotron facilities. In the case of fitting NFS spectra, the first 20 terms of a series expansion are used (Sergueev, 2003 ▸). In NFS spectra the intensity exponentially decreases with time, which is why a user can choose between fitting on a linear scale and on a logarithmic scale which will give a much worse χ^2^ but better fitting of features at a later time where the statistics are worse.

## 4. Manipulations with data and model

*SYNCmoss* can open spectra as files of types *.txt and *.dat which should consist of two columns, one for the velocity and the other for counts. Software can calibrate the velocity scale using the spectrum of α-Fe (*.mca file directly from a CANBERRA multichannel analyzer, where counts are written per channel). Then all other *.mca files will be open with the found velocity scale and can be converted to *.dat files with two columns within the software.

The fitting model can include an arbitrary number of submodels with distributions and correlations. While setting the model one can fix some parameters, link parameters between each other with multiplier coefficients, and set boundaries. There is also the possibility to set an expression as a function of parameters and link other parameters to this expression.

Any minimization method is sensitive to the initial guess of parameters which is why it is necessary to set the initial parameters values somewhere around the expected values. To visualize the model with the initial set of parameters the user can view it by pressing a corresponding button ‘Show model’ (see Fig. 3 ▸) and then correct the parameters if needed, before starting the fitting procedure. All chosen submodels and their starting parameters form the model which can be saved as a text file (*.mdl) to open in the next sessions. After fitting is completed, the result can be saved as an image and text files consisting of a table of parameters and a table containing the spectrum, full model and all submodel envelopes at points where the spectrum is defined (for plotting in any external graphical editor). All fitting and modeling manipulations are performed within the GUI (see Fig. 3 ▸).

An additional feature of the software is the ability to fit spectra in a sequence. If more than one spectrum is selected, then spectra will be fitted one after another. The initial set of parameters for each subsequent spectrum at the discretion of the user can be either the first initial set of parameters or the result of fitting of a previous spectrum in the sequence. This opportunity is extremely useful while fitting data of *operando* and *in situ* measurements or any other big set of similar or slowly changing spectra.

## 5. Mathematical and program details

The task of spectrum fitting always comes down to a minimization problem. The Levenberg–Marquardt algorithm is used to minimize χ^2^ in order to find optimal model parameters [the implementation of the algorithm is given, for example, by Lourakis (2005 ▸)]. This algorithm requires a calculation of first- and second-order partial derivatives. Second-order derivatives are approximated as a direct multiplication of the first-order derivatives. A covariance matrix is estimated at the end of fitting, and is then used to calculate the standard deviation of the model parameters. The Levenberg–Marquardt algorithm in the original form does not accept boundaries for variables. Therefore, the algorithm was slightly modified in such a way that if some parameter during minimization reaches its boundary it becomes fixed at this boundary and further fitting is continued as it is fixed. After optimal parameters are found, there is an attempt to unfix this parameter to check whether it should be change to an allowed direction. The same algorithm is also used while extracting the instrumental function and in the calibration procedure.

As mentioned above, the instrumental function is found as a set of Gaussian lines from a spectrum of the standard absorber. The number of lines can be chosen by the user, and depends on the quality of the standard absorber spectrum and the accuracy needed to fit the experimental data. In order to reduce the calculation time the instrumental function can be refined, and then a previously found instrumental function is used as the initial guess.

Resonance lines in Mössbauer spectroscopy in an ideal case should have Lorentzian shape, but inhomogeneity of Fe nuclei local states (induced by strains, vacancies, dopants, impurities and other defects) leads to additional Gaussian broadening. Therefore, the shape of the resonance line is described by the Voigt function. The Voigt function requires an integral to be calculated that makes this function inconvenient due to the long time needed to reach a sufficient accuracy. Thus, it is better to use an approximation of the Voigt function. In this software the pseudo-Voigt function from Ida *et al.* (2000 ▸) is used as a reasonable compromise between calculation time and accuracy (the deviation from Voigt is ≲0.12%). Two parameters are enough to define the pseudo-Voigt (as well as the Voigt) function. These parameters could be chosen differently; in this software they represent the widths of Lorentzian and Gaussian functions, the convolution of which is the desired Voigt function. The full line width of the intrinsic spectrum could be easily calculated afterwards with an accuracy of 0.01% by using the approximation from Olivero & Longbothum (1977 ▸) if needed.

The experimental spectrum can be described by the full transmission integral [see, for example, Gütlich *et al.* (2011 ▸)] as a convolution of the source instrumental function and absorber intrinsic spectrum. The intrinsic spectrum (which is also called the ‘absorber response’) is the theoretical spectrum of an absorber in the case where the instrumental function is equal to the Dirac delta function. The intrinsic spectrum *S*_in_ can be written as 

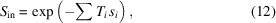

where *T*_*i*_ is the effective thickness of the *i*th component and *s*_*i*_ is a subspectrum of the *i*th component. In the SMS case, knowing that the instrumental function is described by a set of Gaussians, and each subspectrum can be expressed as a set (or distribution) of lines with a Voigt shape, the transmission integral *I*_tr_ could be written as 

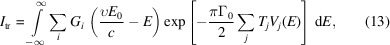

where υ is the velocity of the source relative to the absorber, *E*_0_ is a resonance energy, *c* is the speed of light, πΓ_0_/2 is a normalization constant, and Γ_0_ is the natural width. In the case of a thin absorber it can be calculated easily but the high value of the effective thickness is usually necessary to measure spectra with a high Mössbauer effect to reach the best signal-to-noise ratio within a specific time. Therefore, to take into account the saturation effect, the integral should be calculated in the general form.

For the convolution a numerical integration is used. In order to simplify calculations and to reduce numerical errors we do the following. The integration over energy in infinity boundaries is substituted by the integration from −1 to 1 by a change of the variable to the hyperbolic tangent of energy.^7^ The real boundaries are set to −0.9999 and 0.9999 due to the function not being defined at the boundaries but tending to zero; thus in a numerical case its impact also tends to zero. In order to optimize the integration, the *X*-axis is stretched and *X*-zero moved to be close to the centroid of the instrumental function. The exact stretching coefficient and shifting are calculated to reach minimum error for the specific instrumental function after it is found. From real and simulated cases, it was found that after these manipulations 32 points for the SMS case are sufficient to obtain an accuracy better then 0.1% in most cases. However, in order to reduce the calculation time or to obtain a better accuracy, the number of points per integral could be changed. It is important to mention that the number of points also influences the determination of the instrumental function because this procedure also includes a calculation of the full transmission integral. The calculation of the full transmission integral is the most time-consuming procedure. Thereby it is done in parallel where each process is calculating one point of the integral.

To be sure about the correctness of the model one can rely on χ^2^ and the absence of a systematic deviation of the residual line which is presented in the GUI under the fitted spectrum (Fig. 3 ▸). In order to check the numerical integration accuracy, there is also a line showing the difference between the final model and that calculated with four times more points per integral (Fig. 3 ▸). This line should be straight; if it is curved then the number of points should be increased.

## 6. Conclusions

*SYNCmoss* is a software package designed to fit data obtained with a synchrotron Mössbauer source. The software has a convenient and user-friendly graphical user interface where one can set the initial parameters, fit spectra and save results. The operation of a SMS provides an opportunity to vary the conditions in the range from low resolution and high intensity to vice versa. This leads to a significant change in the shape of the SMS instrumental function. *SYNCmoss* software extracts the instrumental function from a spectrum of the standard sample; then the instrumental function is used in fitting procedures. The software provides a comprehensive functionality to fit data within various models of the hyperfine interaction including the full Hamiltonian case. Among non-common features, it has the ability to set the analytical density probability function for the parameter distribution. Furthermore, the software has an automatic mode to fit a large set of similar spectra in a row. The *SYNCmoss* package consists of code blocks which make it easy to add new mathematical models for other cases of hyperfine interaction. At the time of publishing, this software is already available for users of the ESRF nuclear resonance beamline and later will be distributed by the corresponding author on reasonable request.

## Footnotes

1 The intrinsic spectrum of the standard absorber is a single line with a small distribution of an effective thickness, whose mean value and width along with central shift and resonance line FWHM are stored in an internal file. Thus, if *SYNCmoss* is to be used on other facilities, these parameters can be easily changed to match a specific standard sample.

2 Trying to vary parameters around correct values results in an increase of χ^2^ up to 11.5 which is even worse. Thus, in a real case such a fitting would be rejected due to a worse χ^2^.

3 In the CMS case, an angular distribution answers the question: ‘what is the amplitude of radiation *propagated* along a specific direction?’, and, in the SMS case: ‘what is the amplitude of radiation *polarized* along a specific direction?’.

4 It imposes a requirement that the spectrum should reach the baseline at the edges.

5 In the case of CMS,  =  :  :  , where γ_*k*_ is the angle between a hyperfine field and the direction of the radiation

6 It should be noted that the visible quadrupole splitting depends on θ but the described case is based on a sextet which assumes the quadrupole interaction to be small.

7 In conventional spectroscopy an instrumental function has a Voigt shape with longer tails compared with the SMS case. Thus, while fitting conventional spectra the power series of tangents is used because it results in lower error by slightly increasing the computation time in this case.
